# Thyroid Cytopathology Reporting by the Bethesda System: A Two-Year Prospective Study in an Academic Institution

**DOI:** 10.1155/2015/240505

**Published:** 2015-01-22

**Authors:** Payal Mehra, Anand Kumar Verma

**Affiliations:** Department Of Pathology, Employees State Insurance (ESI) Postgraduate Institute of Medical Sciences and Research and ESI Model Hospital, Basai Darapur, New Delhi 110015, India

## Abstract

*Background*. The Bethesda System for Reporting Thyroid Cytopathology (TBSRTC) has attempted to standardize reporting and cytological criteria in aspiration smears. *Aims*. The objective of this study was to analyze the thyroid cytology smears by TBSRTC, to determine the distribution of diagnostic categories and subcategories, to analyze cytological features, and to correlate the cytopathology with histopathology, wherever surgery was done. *Materials and Methods*. This was a prospective study of 225 fine needle aspirations (FNA) of thyroid nodules. All fine needle aspiration cytology (FNAC) diagnoses were classified according to the features given in the monograph of TBSRTC into nondiagnostic/unsatisfactory (ND/UNS), benign, atypia of undetermined significance/follicular lesion of undetermined significance (AUS/FLUS), follicular neoplasm/suspicious of a follicular neoplasm (FN/SFN), suspicious for malignancy (SFM), and malignant. Cytohistological correlation was done, when surgical material was available. *Results*. The distribution of various categories from 225 evaluated thyroid nodules was as follows: 7.2% ND/UNS, 80.0% benign, 4.9% AUS/FLUS, 2.2% FN, 3.5% SFM, and 2.2% malignant. Sensitivity, specificity, positive predictive value, and negative predictive value were calculated. *Conclusions*. TBSRTC is an excellent reporting system for thyroid FNA. It also provides clear management guidelines to clinicians to go for follow-up FNA or surgery and also the extent of surgery.

## 1. Introduction

Fine needle aspiration cytology (FNAC) of thyroid occupies an extremely important role worldwide. This minimally invasive and cost-effective technique is extremely useful in identifying a substantial proportion of thyroid nodules as benign and reducing unnecessary surgery for patients with benign disease.

However, terminology of reporting thyroid FNACs has varied markedly. Various reporting formats of thyroid FNACs have been used varying from two category schemes to six or more category schemes [1]. While some of them tried to diagnose various lesions using histology-equivalent categories, other formats had categories like equivocal, inconclusive, indeterminate, atypical, suspicious, uncertain, malignancy suspicious, possibly neoplastic, possibly malignant, and probably malignant to report thyroid aspirates that fell between benign and malignant diagnostic categories [1]. It made it difficult for clinicians to interpret the reports. To address terminology and other issues related to thyroid FNACs, the National Cancer Institute (NCI) hosted “The NCI Thyroid Fine Needle Aspiration State of the Science Conference” at Bethesda, Maryland. There were six committees which dealt with different areas regarding thyroid cytology. Committee IV dealt with diagnostic terminology and morphologic criteria for cytological diagnosis of thyroid lesions. Its recommendations were widely published [2, 3]. Subsequently a monograph “The Bethesda System for Reporting Thyroid Cytopathology” (TBSRTC) which includes definitions, diagnostic/morphologic criteria, explanatory notes, and a brief management plan for each diagnostic category was published [4]. TBSRTC is a six-category scheme of thyroid cytopathology reporting (Table 1). Each category has an implied cancer risk, which ranges from 0% to 3% for the “benign” category to virtually 100% for the “malignant” category. It uses three categories, AUS/FLUS, SFN/Hürthle cell neoplasm, and SFM, to report thyroid aspirates that fall between benign and malignant. As a function of these risk associations, each category is linked to evidence based clinical management guidelines.

The objective of the present prospective study, done in an Indian hospital, was to report thyroid cytology smears by TBSRTC into various diagnostic categories, analyze their cytological features using TBSRTC monograph, conveying brief management plan to the clinicians, and correlate with histology of surgical specimens received.

## 2. Materials and Methods

This was a prospective study of all successive cases with thyroid swelling referred to the Department of Pathology, Employees State Insurance (ESI) Postgraduate Institute of Medical Sciences and Research and ESI Model Hospital, Basaidarapur, New Delhi, for FNAC during the period from March 2010 to February 2012. Relevant clinical history was taken and examination done. Pre-FNAC requirements as recommended by Committee I of the NCI State of the Science Conference, Bethesda, were followed [5]. All patients were subjected to FNA sampling under ultrasound guidance by one of the two authors using Zajdela technique 4-5 times randomly in different areas [6]. The smears were prepared using conventional methods and stained with Giemsa and Papanicolaou stains. The cytological features were evaluated and the reporting was done according to TBSRTC (Table 1). The morphological criteria given in the monograph of TBSRTC were used [4]. The clinicians were communicated implied risk of malignancy and recommended clinical management along with the report. Histopathological specimens, wherever available, were processed as per standard methods. Sensitivity, specificity, positive predictive value, and negative predictive value were calculated using histopathology diagnosis as gold standard. For calculating statistical parameters ND/UNS and AUS/FLUS cases were excluded as nondefinitive diagnosis and categories “SFM” and “malignant” were put together. All the parameters were calculated either excluding FN/SFN or including it with either benign or malignant diagnosis to highlight the effect on statistical values.

## 3. Results

The distribution of 225 cases is shown in Table 2. Benign category was the largest (80%) followed by ND/UNS category (7.2%). Malignant and SFM categories constituted 2.2% and 3.6%, respectively, making a total of 5.7%. AUS/FLUS constituted 4.9% cases, while FN/SFN had 2.2% cases.

In the ND/UNS category, all cases were subcategorized as cyst fluid only. There was no case in subcategory virtually acellular specimen or other (obscuring blood, clotting artifact, etc.).

**Figure 1:**
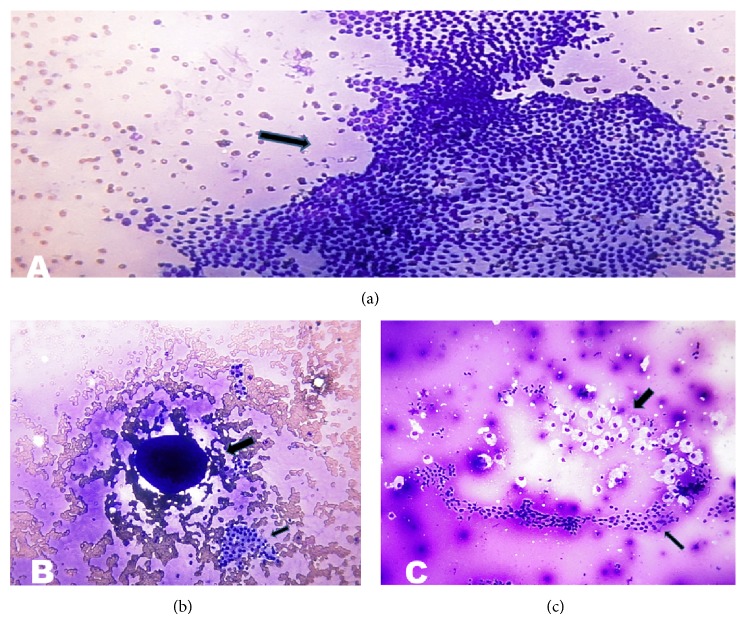
(a) Benign follicular nodule. Photomicrograph showing monolayer sheets of evenly spaced follicular cells having a honeycomb-like arrangement (arrow) (Smear, Giemsa, 400x magnification). (b) Benign follicular nodule. Photomicrograph showing globular mass of colloid with superimposed follicular cells (thick arrow) mixed with monolayer sheet of follicular cells (thin arrow) against the background of colloid and blood (Smear, Giemsa, 400x magnification). (c) Benign follicular nodule. Photomicrograph showing follicular cells arranged in sheets (honeycomb-like) (thin arrow) mixed with macrophages (thick arrow) against the background of colloid (Smear, Giemsa, 400x magnification).

In benign category 76.7% of total cases were consistent with benign follicular nodule (BFN). It had follicular cells which were arranged predominantly in monolayer sheets or were occasionally in intact, 3-dimensional, variably sized balls/spheres (Figures 1(a) and 1(b)). Rare microfollicles were present. Anisonucleosis was seen in some cases but there was no significant pleomorphism or nuclear atypia. Cellularity was low to moderate; low cellularity was seen in 52.2% cases and moderate cellularity in 47.8% cases. High cellularity was not seen. Pleomorphism was present only in 2.9% cases out of 138 cases. Hürthle cells were present only in 2.2% cases. Foam cells (macrophages) were present in 21.7% cases (Figure 1(c)).

Background was blood-mixed with colloid in 55.8% cases and was only colloid in 44.2% cases. Fire flare or spindle cell was not seen in any case of benign follicular nodule in this study.

**Figure 2:**
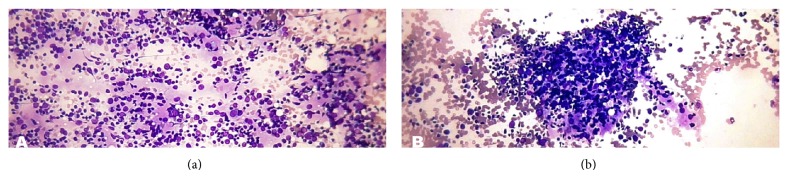
(a) Lymphocytic (Hashimoto) thyroiditis. Photomicrograph showing polymorphous lymphoid population (Smear, Giemsa, 400x magnification). (b) Lymphocytic (Hashimoto) thyroiditis. Photomicrograph showing lymphohistiocytic aggregates in lymphocytic (Hashimoto) thyroiditis (Smear, Giemsa, 400x magnification).

The subcategory consistent with lymphocytic thyroiditis (LT) had 20% cases in benign category. All specimens were moderately cellular. The lymphoid population was moderate in amount in 88.9% cases. The lymphoid cells were polymorphic (Figure 2(a)). Intact lymphoid follicles and lymphohistiocytic (Figure 2(b)) aggregates were also seen. Hürthle cells (oncocytes) were present in all cases. Multinucleated giant cells were found in 27.8% cases and epithelioid cells in 11.1% cases.

**Figure 3:**
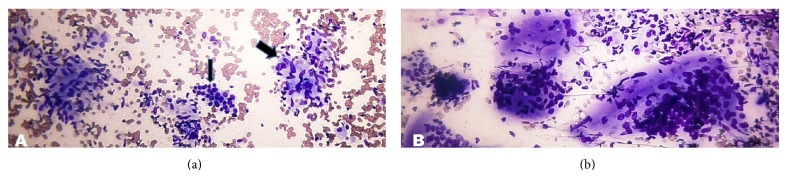
(a) Granulomatous (subacute) thyroiditis. Photomicrograph showing clusters of epithelioid histiocytes (thick arrow) mixed with benign follicular cells (thin arrow) (Smear, Giemsa, 400x magnification). (b) Granulomatous (subacute) thyroiditis. Photomicrograph showing many multinucleated giant cells against the background of colloid and blood (Smear, Giemsa, 400x magnification).

In subcategory consistent with granulomatous thyroiditis (GT), there were 1.1% cases, which showed hypocellular smears with clusters of epithelioid histiocytes (Figure 3(a)), that is, granulomas along with many multinucleated giant cells (Figure 3(b)), lymphocytes, macrophages, and scant degenerated follicular cells. Neutrophils were present in both cases.

The subcategory “other” included cases of chronic abscess. The pus obtained was negative for acid fast bacilli and fungus.

**Figure 4:**
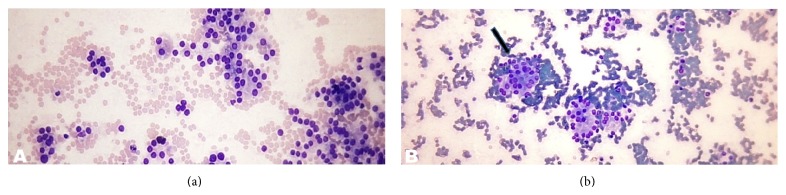
(a) Atypia of undetermined significance. Photomicrograph showing prominent microfollicles in a moderately cellular specimen (Smear, Giemsa, 400x magnification). (b) Atypia of undetermined significance. Photomicrograph showing sparsely cellular specimen with a predominance of microfollicles (Smear, Giemsa, 400x magnification).

In this study, category AUS/FLUS constituted 4.9%. 81.8% of these prominently showed microfollicles in some but not all the moderately cellular smears (Figure 4(a)), 9.1% prominently showed microfollicles with sparsely cellular smear with scant colloid (Figure 4(b)), and 9.1% showed predominantly benign appearing smear with focal features of papillary thyroid carcinoma (PTC) including nuclear grooves, enlarged nuclei with pale chromatin, and alterations in nuclear contour and shape.

**Figure 5:**
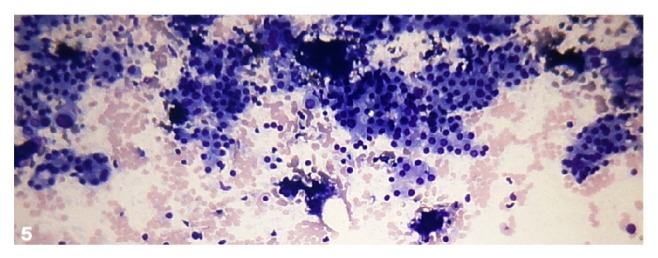
Follicular neoplasm/suspicious for a follicular neoplasm. Photomicrograph showing a highly cellular aspirate composed of uniform follicular cells arranged in crowded clusters and microfollicles (Smear, Giemsa, 400x magnification).

There were 2.2% cases in category FN/SFN (Figure 5). There was no case of FN, Hürthle cell type.

**Figure 6:**
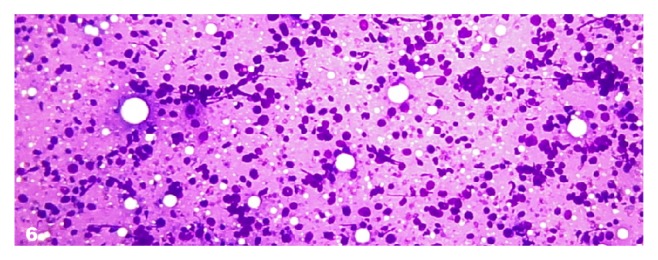
Suspicious for lymphoma. Photomicrograph showing a hemodiluted sample comprising exclusively lymphoid cells (Smear, Giemsa, 400x magnification).

In the category SFM, 75% were suspicious for papillary carcinoma and 12.5% were suspected for lymphoma and 12.5% were SFM, not otherwise specified (SFM, NOS). 66.6% cases of suspicious for papillary carcinoma were of Pattern A (patchy nuclear change), 16.7% cases were of Pattern B (incomplete nuclear change) and 16.7% cases were of Pattern D (cystic degeneration). There was no case of Pattern C (sparsely cellular specimen). 1 (12.5%) case of subcategory suspicious for lymphoma cytologically showed cellular smear composed of numerous monomorphic small to intermediate sized lymphoid cells (Figure 6). In the subcategory SFM, NOS, there was only one case showing cytological features suggestive of malignancy, but they were not enough to categorize the type of malignancy.

**Figure 7:**
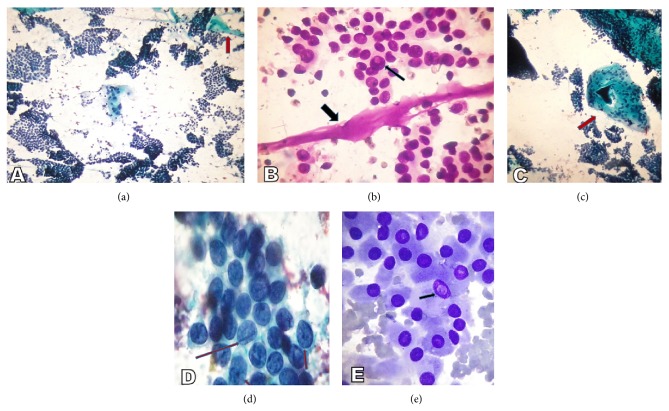
(a) Papillary thyroid carcinoma. Photomicrograph showing highly cellular specimen composed of numerous monolayer sheets and occasional papillary-like fragments and “stringy,” “ropy” colloid (arrow) (Smear, Papanicolaou stain, 400x magnification). (b) Papillary thyroid carcinoma. Photomicrograph showing “stringy” colloid (thick arrow) and intranuclear cytoplasmic inclusions (thin arrow) (Smear, Giemsa, 1000x magnification). (c) Papillary thyroid carcinoma. Photomicrograph showing multinucleated giant cell engulfing sticky colloid (arrow) in a case of papillary thyroid carcinoma (Smear, Papanicolaou stain, 400x magnification). (d) Papillary thyroid carcinoma. Photomicrograph showing longitudinal nuclear grooves (thin long arrow) and micronucleoli (thin short arrows) (Smear, Papanicolaou stain, 1000x magnification). (e) Papillary thyroid carcinoma, oncocytic variant. Photomicrograph showing the neoplasm composed throughout of oncocytic (Hürthle-like) cells that have abundant granular cytoplasm. Intranuclear cytoplasmic inclusions are visible (arrow) (Smear, Giemsa, 1000x magnification).

**Figure 8:**
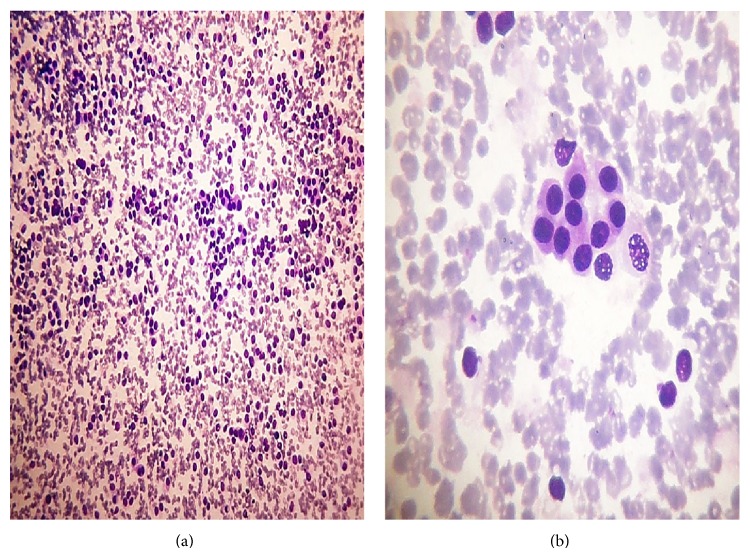
(a) (100x magnification) and (b) (1000x magnification) Medullary thyroid carcinoma. Photomicrographs showing predominantly cohesive, syncytial-like clusters with few isolated plasmacytoid cells (Smear, Giemsa stain).

Category malignant included, 5(2.2%) cases. The maximum number of cases 4 (80%) were of PTC (Figures 7(a)–7(e)) and 1 (20%) case of medullary thyroid carcinoma (MTC) (Figures 8(a) and 8(b)).

Out of 225 cases that were cytologically studied, histopathological specimens of 40 (17.8%) cases were received and studied (Table 3).

Surgical specimens of 2 cases, 23 cases, and 1 case, respectively, for categories ND/UNS, benign, and AUS/FLUS amounting to 12.5%, 12.8%, and 9% of cases aspirated were received.

Of 18 cases of FN/SFN, SFM, and malignant category, only 15 were surgically resected. Out of 4 cases of FN/SFN, 3 were benign (follicular adenoma) and 1 was malignant (PTC). Histopathology was received for 6 cases of SFM. Five of them were reported cytologically as being suspicious for papillary carcinoma. Histopathologically, 3 of them turned out to be papillary thyroid carcinoma, but 2 were lymphocytic (Hashimoto) thyroiditis.

In total, 27 out of 40, that is, 67.5%, cases in this study with a subsequent tissue diagnosis had a definitive cytologic diagnosis of being benign or malignant (Table 4). Twenty cases were benign by both cytopathology and histopathology. Four cases were malignant by both cytopathology and histopathology. None of the cases with a malignant diagnosis on cytology proved to be benign on biopsy and 3 cases out of 23 that were benign on cytology proved to be malignant lesion on examination of tissue specimen.

The results of various statistical parameters are summarized in Table 5.

If FN/SFN is included in malignant group, the sensitivity increases but the specificity decreases. There is marked decrease in positive predictive value also.

## 4. Discussion

This paper shows the two-year experience in reporting thyroid aspirations by TBSRTC in an Indian academic institution as well as response of clinicians to the brief management plan suggested. TBSRTC does not recommend surgery for ND/UNS, benign and AUS/FLUS categories. In the FN/SFN, SFM, and malignant categories, we expected excision of nodules or partial/complete thyroidectomy in all cases as per TBSRTC recommendations.

The present study had 16 (7.2%) cases in ND/UNS category. Other recent studies had 1.2% to 16.4% cases in this group [7–14]. The guidelines for this category are very clear in TBSRTC. All of them were advised to be reaspirated after a minimum period of 3 months. The 3-month interval was recommended to prevent false positive interpretations due to reactive or reparative changes, as recommended by Committee VI (Post-FNA Technique and Treatment Options) [15]. The number of cases in this category is dependent on the aspirator's experience. The recommendations of Committee I on indications of thyroid FNA and pre-FNA and Committee II on training and credentialing are likely to bring down the number in this category in future studies [5, 16]. TBSRTC does not provide the implied risk of malignancy for this category. However a recent study found a rate of 8.9% in ND/UNS category [10]. Two histopathological specimens were received and both were nodular goiter. Clinician was not comfortable with the term ND/UNS and was not willing to wait for 3 months, thus preferred to go for surgery.

The benign category had 180 cases (80%) with BFN being the predominant group followed by LT. The “benign” category had a range of 34% to 87.5% in recent studies [7–14]. However, only one study had a percentage less than 50 and this was due a high incidence of AUS/FLUS and FN/SFN. The diagnostic criteria of all the subcategories are well characterized in TBSRTC monograph. However, TBSRTC recommendation on diagnostic terminology and morphologic criteria does not mention giant cells and epithelioid cells in LT previously described in the literature [17, 18]. 23 histopathological specimens from category diagnosed as “benign” were received. All of them were operated because of cosmetic reasons or pressure symptoms. 18 were nodular goiter, 2 follicular adenoma, and 3 PTC. The cytological appearance of nodular goiter can overlap with those of follicular adenoma and cytological criteria alone cannot reliably distinguish between the two in certain cases [19]. These 2 cases had on cytology abundant colloid in addition to follicular cells and hence were diagnosed as BFN. There were 3 cases of PTC which were incidental findings in thyroid specimen and were less than 1 cm in size and thus these nodules were not aspirated. There were no lymph nodes in these cases and ultrasound features were not suspicious. These “incidentalomas” remain indolent in most cases as implied by 30% prevalence in a study by Harach et al. in 1985 in an autopsy study [20]. One study does not consider incidental papillary carcinoma as malignant for analysis of their data [10].

The classification of “indeterminate” lesions (those not clearly benign or malignant) in thyroid cytopathology has long been a source of confusion for both pathologists and clinicians. The general category AUS/FLUS is reserved for specimens that contained cells (follicular, lymphoid, or other) with architectural and/or nuclear atypia that is not sufficient to be classified as suspicious for a follicular neoplasm or suspicious for malignancy. The atypia is more marked than can be ascribed confidently to benign changes.

We had 11 cases in group AUS/FLUS. An AUS result has been reported in 3.2–29% of thyroid cases [7–14]. TBSRTC suggests that the frequency of AUS interpretations should be in the range of approximately 7% of all thyroid FNA interpretations. This is a category of last resort and should not be used indiscriminately. Not much data exists in the literature to support the recommendation that the category should not exceed 7% of all thyroid categories [21]. The incidence also varies with experience and training of cytopathologists. The recommended management for an initial AUS/FLUS interpretation is the clinical correlation and, for most cases, a repeat FNA at an appropriate interval. A repeat FNA usually results in a more definitive interpretation; only about 20–25% of nodules are repeatedly AUS. One case of AUS/FLUS on cytology proved to be malignant on histology and was PTC. On cytology, this case showed predominantly benign appearing smear with focal features of PTC in only one of the smears and thus was put under this category.

FN/SFN category had 8 cases (2.2%). TBSRTC provides clear guidelines for this category. Recent studies have shown 2.2–16.1% cases in this group [7–14]. Four specimens were received, 1 of which was follicular variant of papillary carcinoma. Smears of the latter had predominant follicular pattern but the classic nuclear features of PTC were not present in the cytological smears.

SFM category had 8 cases (3.6%), 6 of which were suspicious for papillary carcinoma, one was suspicious for lymphoma, and one was SFM, NOS. Histopathology was received for 6 cases of SFM. It varies from 1.3 to 10% in recent studies [7–14]. Five of them were reported cytologically as suspicious for papillary carcinoma. Histopathologically 3 of them turned out to be papillary thyroid carcinoma, but 2 were lymphocytic (Hashimoto) thyroiditis. The latter were on cytology diagnosed as suspicious for papillary carcinoma because of high proportion of follicular cells and presence of intranuclear cytoplasmic inclusions (INCIs) in rare cells. INCIs are not specific for papillary thyroid carcinoma as they may be seen focally in benign thyroid nodules. Moreover, an increased incidence of PTC is well known in LT and hence a diagnosis of suspicious for papillary carcinoma was given so as not to miss out malignancy. SFM, NOS, had very high cellularity with 3D clusters of follicular cells in all the smears but could not be typed cytologically. Histologically, it turned out to be follicular adenoma.

Committee V of the NCI Thyroid Fine Needle Aspiration State of the Science Conference has provided guidelines for indications of ancillary studies, specific ancillary studies to be performed, and sample preparation for each study. Immunohistochemistry panels have been suggested for suspicious malignancies which include medullary carcinoma (calcitonin, thyroglobulin, CEA, and chromogranin), anaplastic carcinoma (pan-cytokeratin), and metastatic carcinoma (TTF-1). These are to be done on cell block from FNA, preferably including at least one dedicated pass for the study. For suspicious lymphoma, flow cytometric immunophenotyping is suggested. Dedicated passes are also needed for studies to detect genetic alterations such as BRAF mutation or RET/PTC chromosomal rearrangements, which are very promising for the diagnosis of papillary carcinoma. Immunocytochemistry on cytospin, direct smear, or prefixed monolayer may also be utilized, but protocols should be carefully validated [22].

The category malignant had a range of 2.9% to 11% in recent studies [7–14]. The present study had 5 (2.2%) cases in the malignant category. We received 4 specimens from the category diagnosed as “malignant” cytologically. All of them were diagnosed as PTC both histopathologically and cytologically.

Table 6 shows a comparison of statistical parameters of our study and other studies over the last 30 years.

The method of data analysis can alter the results of statistical parameters. If suspicious lesions are considered positive, the sensitivity increases while the specificity decreases. If suspicious lesions are excluded, then the sensitivity decreases and the false negative rates increase. For statistical purpose we had put categories “SFM” and “malignant” in one group. “Unsatisfactory” smears were likely to be malignant or benign and putting them into a single diagnostic category automatically presents a false picture. All the parameters were calculated either excluding FN/SFN or including it with either benign or malignant diagnosis to highlight the effect on statistical values.

Articles implementing TBSRTC have started appearing in the literature [14, 23–25]. TBSRTC is a relatively recent six-category scheme to classify thyroid cytology smears. It needs to be validated by more prospective studies on larger number of cases with histopathological correlation. There is need for consensus amongst institutions in various countries to utilize TBSRTC to facilitate easy sharing of data across the world for surveys and research.

## 5. Conclusions

This study is a prospective analysis of reporting of thyroid aspiration smears by TBSRTC using the Bethesda monograph. It was found that the monograph is succinctly written in an easy-to-read format and has useful color images which help in making the diagnosis. The clinicians are also benefitted because of the management plan it suggests. Cases of AUS/FLUS are followed by repeat FNAC, thus reducing the incidence of surgery in our series (1/11). However, the exact incidence of malignancy in this heterogeneous category is difficult to predict as most of these cases are unlikely to be operated if the Bethesda recommendations are to be followed. There is a need for a large study with histopathological correlation for this category.

## Figures and Tables

**Table 1:** The Bethesda System for reporting thyroid cytopathology: recommended diagnostic categories, implied risk of malignancy, and recommended clinical management.

Diagnostic category	Risk of malignancy (%)	Usual management^a^
**(I) Nondiagnostic or unsatisfactory (ND/UNS)**		Repeat FNA with ultrasound guidance
Cyst fluid only		
Virtually acellular specimen		
Other (obscuring blood, clotting artifact, etc.)		
**(II) Benign**	0–3	Clinical follow-up
Consistent with a benign follicular nodule (includes adenomatoid nodule,colloid nodule etc.)		
Consistent with lymphocytic (Hashimoto) thyroiditis in the proper clinical context		
Consistent with granulomatous (subacute) thyroiditis		
Other		
**(III) Atypia of undetermined significance or follicular lesion of undetermined significance (AUS/FLUS)**	5–15^b^	Repeat FNA
**(IV) Follicular neoplasm or suspicious for follicular neoplasm (FN/SFN)**	15–30	Surgical lobectomy
-specify if Hürthle cell (oncocytic) type		
**(V) Suspicious for malignancy (SFM)**	60–75	Near-total thyroidectomy or surgical lobectomy^c^
Suspicious for papillary carcinoma		
Suspicious for medullary carcinoma		
Suspicious for metastatic carcinoma		
Suspicious for lymphoma		
Other		
**(VI) Malignant**	97–99	Near-total thyroidectomy^c^
Papillary thyroid carcinoma		
Poorly differentiated carcinoma		
Medullary thyroid carcinoma		
Undifferentiated (anaplastic) carcinoma		
Squamous cell carcinoma		
Carcinoma with mixed features (specify)		
Metastatic carcinoma		
Non-Hodgkin lymphoma		
Other		

^a^Actual management may depend on other factors (e.g., clinical and sonographic) besides the FNA interpretation.

^b^Estimate extrapolated from histopathologic data from patients with “repeated atypicals” [7, 26].

^c^In the case of “suspicious for metastatic tumor” or a “malignant” interpretation indicating metastatic tumor rather than a primary thyroid malignancy, surgery may not be indicated.

**Table 2:** Number of cases in various diagnostic categories and subcategories according to the Bethesda System for Reporting Thyroid Cytopathology (TBSRTC).

S. number	Cytological categories	Subcategories	Number of cases	Total number of cases in each category
		Cyst fluid only	16	
1	Nondiagnostic/unsatisfactory (ND/UNS)	Virtually acellular specimen	0	16 (7.2)
		Other (obscuring blood, clotting artifact, etc.)	0	

		Consistent with benign follicular nodule (includes adenomatoid nodule, colloid nodule, etc.)	138	
2	Benign	Consistent with lymphocytic (Hashimoto) thyroiditis in the proper clinical context	36	180 (80)
		Consistent with granulomatous (subacute) thyroiditis	02	
		Other	04	

3	Atypia of undetermined significance/follicular lesion of undetermined significance (AUS/FLUS)			11 (4.9)

4	Follicular neoplasm/suspicious for a follicular neoplasm (FN/SFN)			5 (2.2)

		Suspicious for papillary carcinoma	06	
		Suspicious for medullary carcinoma	0	
5	Suspicious for malignancy (SFM)	Suspicious for metastatic carcinoma	0	8 (3.6)
		Suspicious for lymphoma	01	
		Other	01	

		Papillary thyroid carcinoma	04	
		Poorly differentiated carcinoma	0	
		Medullary thyroid carcinoma	01	
		Undifferentiated (anaplastic) carcinoma	0	
6	Malignant	Squamous cell carcinoma	0	5 (2.2)
		Carcinoma with mixed features	0	
		Metastatic carcinoma	0	
		Non-Hodgkin lymphoma	0	
		Other	0	

	Total			**225 (100)**

(1) Figures in parentheses indicate percentages.

(2) “Other” subcategory in benign category consisted of cases of chronic nonspecific abscess.

(3) “Other” subcategory in suspicious for malignancy category consisted of a case of suspicious for malignancy, not otherwise specified.

**Table 3:** Cytological/histopathological diagnosis correlation.

Cytopathological categorization	Number of cases where surgical specimens were received (*n* = 40)	Percent of the category	Histopathological diagnosis	Number of cases
ND/UNS (*n* = 16)	2	12.5	Nodular goiter	2

Benign (*n* = 180)			Nodular goiter	18
	23	12.8	Follicular adenoma	2
			Papillary thyroid carcinoma	3

AUS/FLUS (*n* = 11)	1	9	Papillary thyroid carcinoma	1

FN/SFN (*n* = 5)	4		Follicular adenoma	3
		80	Papillary thyroid carcinoma	1

SFM (*n* = 8)	6		Lymphocytic thyroiditis	2
		75	Papillary thyroid carcinoma	3
			Follicular adenoma	1

Malignant (*n* = 5)	4	80	Papillary thyroid carcinoma	4

(1) ND/UNS = nondiagnostic/unsatisfactory, AUS/FLUS = atypia of undetermined significance/follicular lesion of undetermined significance, FN/SFN = follicular neoplasm/suspected for a follicular neoplasm, and SFM = suspected for malignancy.

(2) *n* = total number of cases.

**Table 4:** Cytological/histopathological correlation with benign and malignant cases.

Cytodiagnosis	Histologic diagnosis	
	Benign	Malignant
ND/UNS (*n* = 2)	2	0
Benign (*n* = 23)	20	3
AUS/FLUS (*n* = 1)	0	1
FN/SFN (*n* = 4)	3	1
SFM (*n* = 6)	3	3
Malignant (*n* = 4)	0	4

(1) ND/UNS = nondiagnostic/unsatisfactory, AUS/FLUS = atypia of undetermined significance/follicular lesion of undetermined significance, FN/SFN = follicular neoplasm/suspected for a follicular neoplasm, and SFM = suspected for malignancy.

(2) *n* = total number of cases.

**Table 5:** Statistical parameters when FN/SFN cases are excluded or are included with either benign or malignant cases.

Statistical Parameters	FN/SFN cases excluded	FN/SFN cases included with benign cases (%)	FN/SFN cases included with malignant cases (%)
Sensitivity	76.92 (46.20–94.69)	73.33 (44.91–92.05)	78.57 (49.21–95.09)
Specificity	88.46 (69.82–97.42)	89.66 (72.62–97.69)	81.25 (63.55–92.75)
Positive predictive value	76.92 (46.20–94.69)	78.57 (49.21–95.09)	64.71 (38.35–85.70)
Negative predictive value	88.46 (69.82–97.42)	86.67 (69.26–96.16)	89.66 (72.62–97.69)

Figures in parentheses show 95% confidence interval.

**Table 6:** Comparison of results of the present study and random studies over the last 30 years.

Study	Number	Sensitivity %	Specificity %	PPV %	NPV %
Al-Sayer et al. [27]	70	86	93	80	96
Silverman et al. [28]	309	93	96.5	88.9	96.5
Cusick et al. [29]	283	76	58	72	64
Altavilla et al. [30]	257	71.4	100	100	94.4
Bouvet et al. [31]	78	93.5	75	85.3	88.2
Ko et al. [32]	207	78.4	98.2	99	66.3
Kessler et al. [33]	170	79	98.5	98.7	76.6
Handa et al. [34]	66	97	100	96	100
Gupta et al. [35]	75	80	86.6	80	86.6
Present study (FN/SFN excluded)	40	76.92	88.46	76.92	88.46
Present study (FN/SFN included as benign)	40	73.33	89.66	78.57	86.67
Present study (FN/SFN included as malignant)	40	78.57	81.25	64.71	89.66

PPV = positive predictive value and NPV = negative predictive value.
